# Supplementary material for: Health-related quality of life and emotional and behavioral difficulties after extreme preterm birth: developmental trajectories
Source: PeerJ. 2015 Jan 20;3:e738. doi: 10.7717/peerj.738 (PMC4304859; doi:10.7717/peerj.738)
Supplement: Table S1 [file peerj-03-738-s001.docx]

**Table S1** Clinical, socio-demographic and neonatal characteristics of the extremely preterm (EP) and term-born children

| **Variables** | **EP-born** | | **Term-born** | | | **P-value^a^**  (18 years) |
| --- | --- | --- | --- | --- | --- | --- |
|  | **10 years**  (n = 35) | **18 years**  (n = 31) | **10 years**  (n = 35) | | **18 years**  (n = 29) |  |
| Boys, n (%) | 13 (37) | 13 (42) | 13 (37) | | 9 (31) |  |
| Age at assessment, mean (SD) | 10.5 (0.4) | 17.7 (0.4) | 10.7 (0.4) | | 17.9 (0.5) |  |
| Asthma^b^, n (%) | 10 (29) | 6 (19) | 1 (3) | | 5 (17) | 0.84 |
| Minor disabilities^c^ (%) | 7(20) | 7 (20) | 0 | | 0 |  |
| Mothers’ education below college/university, n (%) | 29 (85)^d^ | 23 (74) | 19 (54) | | 11 (39)^d^ | 0.007 |
| Educational level, n (%):  -vocational upper secondary  -academic upper secondary |  | 18 (58)  13 (42) |  | | 5 (19) ^e^  22 (81) | 0.003 |
| General Self-Efficacy^f^, median (IQR) |  | 3.0 (2.6,3.2) |  | | 3.2 (3.0,3.5) | 0.04 |
| Victim of bullying last months, n (%):  -never/rarely  -monthly/weekly |  | 30 (97)  1 (3) |  | | 28 (97)  1 (3) | 0.96 |
| Active member of an athletic club, n (%) | 18 (51) | 9 (30)^d^ | 26(79)^g^ | | 11 (38) | 0.54 |
| Leisure time physical activity, n (%):  - < 3 times monthly  - 1 to 3 times weekly  - > 4 times weekly |  | 7 (23)  15 (48)  9 (29) |  | | 5 (17)  15 (52)  9(31) | 0.70 |
| Visiting friends last week, n (%):  - none  - 1-3 times  - > 4 times |  | 8 (26)  18 (58)  5 (16) |  | | 1 (3)  23 (79)  5 (17) | 0.03 |
| Receiving friends last week, n (%):  - none  - 1-3 times  - > 4 times |  | 7 (23)  20 (64)  4 (13) |  | | 8 (28) ^d^  17 (61)  3 (11) | 0.40 |
|  | **Neonatal characteristics** | | | | | |
|  | **EP-born**  (n = 35) | | | **Term-born**  (n = 35) | | |
| Birth weight, mean (SD) | 933 (204) | | | 3564 (275) | | |
| Small for gestational age^h^, n (%) | 9 (26) | | | 0 | | |
| Gestational age, mean (SD) | 26.7 (1.7) | | | > 37 | | |
| Intra-ventricular hemorrhage grade 1-2, n (%) (none grade 3-4) | 8 (23) | | | 0 | | |
| Bronchopulmonal dysplasia, n (%)  -none  -mild^i^  -moderate/severe^j^ | 9 (26)  14 (40)  12 (34) | | | 35 (100)  0  0 | | |
| Days on ventilator, mean (SD) | 8.3 (11.8) | | | 0 | | |
| Days on oxygen treatment, mean (SD) | 57.4 (48.0) | | | 0 | | |
| Steroid treatment for BPD, n (%) | 10 (29) | | | 0 | | |

a) From generalized linear model

b) Diagnosed according to wheeze in last 12 month plus either ‘asthma ever’ or asthma medication in last 12 months

**Table S1** “Continued” Clinical, socio-demographic and neonatal characteristics of the extremely preterm (EP) and term-born children

c) Attention Deficit Hyperactivity Disorder, mild mental retardation, epilepsy, impaired hearing (some with comorbidity)

d) One missing

e) Two in the term –born group did not attend school (one pregnant, one working)

f) Scale 1-4, median (25^th^ – 75^th^ percentile), p-value from Wilcoxon’s signed rank test

g) Two missing

h) Birth weight < 10 percentile according to Norwegian growth standards

i) Requirement for oxygen treatment age 28 postnatal days

j) Requirement for oxygen treatment at 36 weeks postmenstrual age
